# Serum Cystatin C Level Is Not a Promising Biomarker for Predicting Clinicopathological Characteristics of Bladder Urothelial Tumors

**DOI:** 10.1155/2018/2617439

**Published:** 2018-01-29

**Authors:** Hui Wang, Lijian Gao, Cuiyu Meng, Nengwang Yu, Feilong Yang, Cong Zhang, Dawei Li, Lei Yan, Hainan Liu, Zhonghua Xu

**Affiliations:** ^1^Department of Urology, Qilu Hospital of Shandong University, Jinan 250012, China; ^2^Department of Urology, Dezhou People's Hospital, Dezhou, Shandong Province 253000, China; ^3^Department of ICU, Dezhou People's Hospital, Dezhou, Shandong Province 253000, China

## Abstract

The role of cystatin C (Cys-C) in tumorigenesis and progression of bladder urothelial tumors (BUT) is still indefinite. We retrospectively collected the clinical information from the records of 425 BUT patients. Pretreatment serum Cys-C levels were compared across the various groups. Then we subgroup the patients with GFR ≥ 90 mg/min/1.73 m^2^, to exclude the effects of lower renal function on cystatin C. No statistically significant differences in the levels of serum Cys-C were found among the tumor characteristics (all *P* > 0.05). In conclusion, circulating Cys-C was not a reliable predictor for clinicopathological characteristics of BUT patients.

## 1. Introduction

By 2020 the estimated incidence of genitourinary (GU) cancers (prostate, bladder, and kidney) will be over 2 million worldwide and responsible for approximately 800,000 deaths [1]. The majority of bladder cancers are transitional cell carcinomas, which account for more than 90% of all cases, while squamous cell carcinoma and adenocarcinoma are less prevalent [2]. Leiomyomas of the bladder constitute <0.5% of all bladder tumors [3]. According to the World Health Organization/International Society of Urological Pathology (WHO/ISUP) classification, bladder urothelial tumors are categorized into three subtypes: papilloma, papillary urothelial neoplasm of low malignant potential (PUNLMP), and carcinoma (bladder urothelial carcinoma, BUC) [4]. Carcinomas are further subcategorized based on the presence of muscular invasion into non-muscle-invasive BUC (NMIBC) (Stage Tis-Ta-T1) and muscle-invasive BUC (MIBC) (Stage T2-T3-T4) [5]. BUC is the ninth most common cancer worldwide. The first economic study of BUC and the first real-life evidence of the current therapeutic algorithm act in the Italian context. The increase in prevalence requires continuous surveillance and care, resulting in a significant burden on Italian National Health Service, making any improvement to the strategy for diagnosing and treating this disease important to the medical and scientific community [6].

In current clinical practice, the diagnoses of BUT rely on sophisticated instruments and skilled personnel. Cystoscopy and biopsy were performed as a gold method. But this approach brings lots of discomfort to the patient (pain, fear, tension, etc.). Cancer markers may offer the advantages of simplicity, accurately and comfortably. Now, a number of markers have been described. But the performance and robustness of these markers remain unclear.

Cystatin C is the potent inhibitor of cysteine proteinases [7]. It is abundant in various body tissues and fluids [8]. Previous studies revealed a correlation between high serum levels of cysteine proteinase inhibitors and poor prognosis in malignant melanoma and colorectal cancer [9]. The association of disturbances in the ratio between cysteine proteinases and endogenous inhibitors of cysteine proteinases with malignant progression is well established.

## 2. Materials and Methods

### 2.1. Patient Population

A review of medical records was performed for 425 patients who were newly diagnosed with BUT and received surgical management at the Department of Urology, Qilu Hospital of Shandong University, between January 2010 and October 2014. Prior to participation, the following criteria were used to exclude patients:

1. Coexisting upper tract urothelial tumors or other tumors
2. History of bladder urothelial tumors and upper tract urothelial tumors
3. History of cerebral infarction and cerebral hemorrhage within the last 1 month or myocardial infarction within the latest 6 months
4. Administration of procoagulant or anticoagulant drugs within the past 2 weeks
5. There is an obvious infection and inflammation.

### 2.2. Data Collection

Clinical data including patient age at the time of diagnosis, sex, smoking history, painless macroscopic hematuria, routine blood examination results (white blood cell count, platelet count, plasma fibrinogen level, etc.), and other tumor characteristics were obtained from the electronic patient records at our institution. Tumor grade was assessed according to the 1998 WHO/ISUP classification, and tumor stage was evaluated using the 2002 TNM classification.

### 2.3. Cys-C Measurement

*Sample Test and Data Collection*. After 8 hours of fasting, 5 mL of venous blood was obtained from patients with BUT and assayed immediately before clinical treatment. Blood samples were deposited into test tubes containing a clot activator and gel. The circulating Cys-C levels were tested with immunoturbidimetric method using a Roche Cobas 8000 analyzer with reagents purchased from BioSino Bio-Technology and Science Inc., Beijing, China. Cys-C levels of 0.51–1.09 mg/L were defined as normal. The level of SCr was determined using a Roche Cobas 8000 analyzer with reagents purchased from Roche. SCr levels of 62–115 *μ*mol/L were defined as normal. The tests were completed according to the manufacturers' instructions. Clinical data, including age, SCr, and Cys-C, were retrieved from patient files.

We first compared pretreatment serum Cys-C levels among all patients. Subsequently, we divided the patients into two groups according to the glomerular filtration rate (GFR < 90 mL/min/1.73 m^2^ and GFR ≥ 90 mL/min/1.73 m^2^). Subclass analyses were conducted to exclude the possible effects of renal function on the pretreatment levels of Cys-C. We also analyzed the association of serum Cys-C levels with clinical characteristics of BUT patients. Linear correlations among age, plasma fibrinogen level, and Cys-C were also evaluated.

We first need to exclude the effect of glomerular filtration rate (GFR) decline on Cys-C. Chronic kidney disease (CKD) was classified into five stages based on the renal glomerular filtration rate (rGFR) values as follows [10]:

  Stage 1, rGFR ≥ 90 mL/min/1.73 m^2^
  Stage 2, 60 mL/min/1.73 m^2^ ≤ rGFR < 90 mL/min/1.73 m^2^
  Stage 3, 30 mL/min/1.73 m^2^ ≤ rGFR < 60 mL/min/1.73 m^2^
  Stage 4, 15 mL/min/1.73 m^2^ ≤ rGFR < 30 mL/min/1.73 m^2^
  Stage 5, rGFR < 15 mL/min/1.73 m^2^.

Renal insufficiency was defined as rGFR < 60 mL/min/1.73 m^2^. Cystatin C is a sensitive index of renal function injury. A mild kidney injury can lead to a change in cystatin C. In many cases, the Cys-C has changed before GFR declined. So rGFR ≥ 90 mL/min/1.73 m^2^ was used for grouping patients. The Modification of Diet in Renal Disease (MDRD) equation, initiated in 1999 and based on the serum creatinine level, is still applied clinically after several modifications [11]. Chinese_MDRD (eGFRc-MDRD), 175 × (Scr)^−1.234^ × (age)^−0.179^  × (×0.79 if female), was used to estimate GFR [12].

### 2.4. Statistical Analysis

The normal distribution of quantitative data in the various groups was assessed by the Kolmogorov-Smirnov test. Normally distributed data were expressed as the mean ± standard deviation (SD), while the median (range) was reported for data not following a Gaussian distribution. Statistical analyses were accordingly performed using the parametric Student's* t*-test and one-way ANOVA. Qualitative data were reported as numbers and percentages. Linear correlation analyses examine the associations among age, plasma fibrinogen levels, and Cys-C levels. Data were analyzed and processed using the Statistical Package for Social Sciences version 20.0 (SPSS Inc., Chicago, IL, USA). All probabilities were two-tailed and values less than 0.05 were considered statistically significant.

## 3. Results

### 3.1. Characteristics of Study Population

The study group consisted of patients with BUT consecutively presenting at the Department of Urology, Qilu Hospital of Shandong University. In total, 425 patients conforming to inclusive criteria were eligible for inclusion in the final study. The mean age of patients was 61.93 ± 13.17 years, ranging from 19 to 96. All patients were further grouped according to their levels of GFR. Clinical characteristics of enrolled patients are presented in Table 1.

The present study included 425 patients with newly diagnosed BUT. Among patients with GFR ≥ 90 mg/min/1.73 m^2^, 263 were men and 58 were women. The mean Cys-C level of this cohort was 0.91 ± 0.15 mg/L. Among patients with GFR < 90 mg/min/1.73 m^2^, 81 were men and 23 were women. The mean Cys-C level of this cohort was 1.32 ± 0.77 mg/L. The mean Cys-C level of patients with GFR ≥ 90 mg/min/1.73 m^2^ was significantly lower than that of patients with GFR < 90 mg/min/1.73 m^2^ (*P* ⩽ 0.001).

### 3.2. Association of Serum Cys-C Levels with Clinical Characteristics of BUT in All Patients Group

We found that the levels of serum Cys-C in elder BUT patients (more than 60 years) were higher than in younger patients (*P* = 0.013). The mean Cys-C level of patients with plasma fibrinogen levels (PFL) < 2.9 g/L was significantly lower than that of patients with PFL ≥ 2.9 g/L (*P* = 0.030). Clinical characteristics of enrolled patients are presented in Table 2.

### 3.3. Association of Serum Cys-C Levels with Clinical Characteristics of BUT

**Figure 1:**
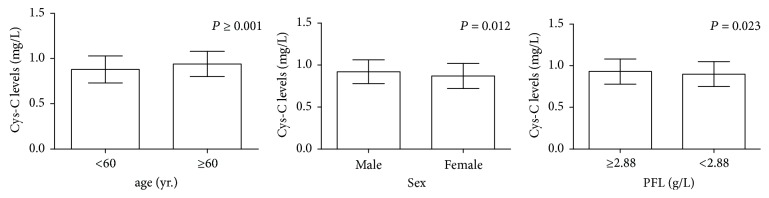
The levels of serum Cys-C in elder BUT patients (more than 60 years) were higher than that in younger patients (*P* ⩽ 0.001). The levels of serum Cys-C in male BUT patients were higher than in female patients (*P* = 0.012). The mean Cys-C level of patients with PFL < 2.88 g/L, which was significantly lower than that of patients with PFL ≥ 2.88 g/L (*P* = 0.023).* P*: parametric Student's *t*-test.

Considering the effect of GFR on levels of serum Cys-C, the associations between serum Cys-C levels and clinical characteristics of 321 patients with normal GFR (≥90 mg/min/1.73 m^2^) were further evaluated. BUT patients were stratified accordingly, and these data are presented in Table 3. We found that the levels of serum Cys-C in elder BUT patients (more than 60 years) were higher than that in younger patients (*P* ⩽ 0.001). The levels of serum Cys-C in male BUT patients were higher than in female patients (*P* = 0.012). The mean Cys-C level of patients with PFL < 2.88 g/L was significantly lower than that of patients with PFL ≥ 2.88 g/L (*P* = 0.023) (Figure 1).

### 3.4. Levels of Serum Cys-C Correlate with Age and PFL in BUT Patients with Normal GFR

**Figure 2:**
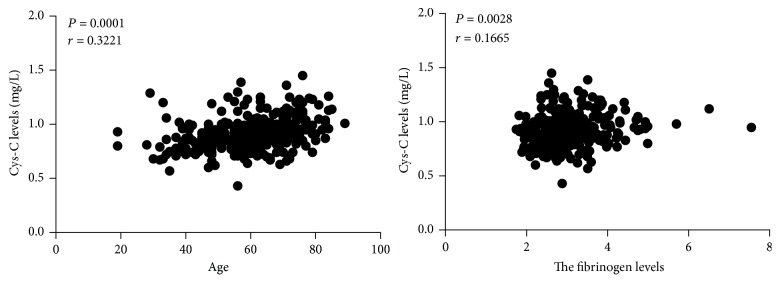
Using linear correlation analyses, there were positive correlations between circulating Cys-C levels, age (*r* = 0.3221, *P* = 0.0001), and PFL (*r* = 0.1665, *P* = 0.028). *P*: statistical significance; *r*: correlation coefficient according to Pearson correlation test.

Using linear correlation analyses, there were positive correlations between circulating Cys-C levels, age (*r* = 0.3221, *P* = 0.0001), and PFL (*r* = 0.1665, *P* = 0.028) (Figure 2).

**Figure 3:**
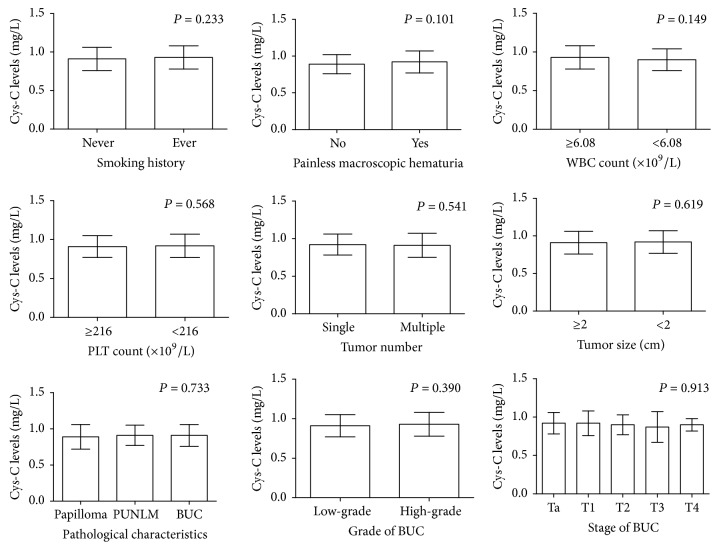
There were insignificant associations between the levels of serum Cys-C and clinical characteristics, such as smoking history, painless macroscopic hematuria, WBC count, PLT count, tumor number, tumor size, and pathological characteristics (all *P* > 0.05). *P*: parametric Student's *t*-test.

Moreover, there were insignificant associations between the levels of serum Cys-C and clinical characteristics, such as smoking history, painless macroscopic hematuria, WBC count, PLT count, tumor number, tumor size, and pathological characteristics (all *P* > 0.05) (Figure 3).

## 4. Discussion

In the present study, we examined preoperative Cys-C levels and clinical features of 425 patients with newly diagnosed BUT. Insignificant associations were observed between tumor characteristics and Cys-C levels (tumor number, tumor size, and pathological characteristics) (all *P* > 0.05). Our results indicate that Cys-C level is not a promising biomarker for predicting pathological outcomes in patients with BUT. To our knowledge, this study is the first to focus on alterations of circulating Cys-C concentrations in patients with BUT using this method. A study published in 2006 indicates that cystatin C concentrations are not directly correlated with the progression of primary bladder carcinomas [13]. The results are consistent with our findings. Our result was in accordance with that from another study focusing on renal cell carcinoma and pancreatic tumors, which excluded the role of serum Cys-C level as possible biomarker [14]. Another study focusing on ovarian cancer indicates the role of serum Cys-C level as possible biomarker [15].

CST3 is located on the short arm of chromosome 20, spans 7.3 kb, contains four exons, encodes a 120-amino-acid active cysteine proteinase inhibitor, and shares several features with housekeeping genes [16]. Cys-C is ubiquitously expressed in nucleated cells [17] in tissues such as the testis, epididymis, seminal vesicle, and prostate [18] and is then secreted into various human fluids to inhibit the activity of cysteine proteases such as papain and cathepsins B, H, K, and L [19].

Cys-C is considered to function as a p53-inducible tumor suppressor and apoptotic mediator that negatively regulates cathepsin L activity during carcinogenesis [20]. Cys-C is believed to play a key role in the tumor suppressive function of p53 [20], as well as in extracellular protein homeostasis. An imbalance between Cys-C and cysteine proteinases has been discovered in the pathogenesis of a broad spectrum of diseases [21]. But the diagnostic role of Cys-C has been dismissed in some cancers, such as renal cell carcinoma and pancreatic tumors [14]. Cys-C was also found to modulate the invasion of prostate cancer cells by means of the androgen receptor and MAPK/Erk2 pathways [22]. Cys-C is also a marker for inflammation [23] and infection [21].

The mean Cys-C level of patients with GFR ≥ 90 mg/min/1.73 m^2^ was significantly lower than that of patients with GFR < 90 mg/min/1.73 m^2^. Cys-C is a cationic, nonglycosylated protein with a molecular mass of 13 kDa. It is ubiquitously expressed in all nucleated cells, widely distributed in human biological fluids [17], freely filtered through renal glomeruli, and uniquely and almost completely reabsorbed and catabolized in the proximal tubules [24]. Therefore, its classic role as a sensitive marker for renal function has been extensively studied [24] and further confirmed in a meta-analysis.

In the whole BUT patients, we found that the levels of serum Cys-C in elder BUT patients (more than 60 years) were higher than in younger patients (*P* = 0.013). The mean Cys-C level of patients with PFL < 2.9 g/L was significantly lower than that of patients with plasma fibrinogen levels ≥ 2.9 g/L (*P* = 0.030). In the subgroup of bladder urothelial tumors patients with normal GFR, we also found that the levels of serum Cys-C in elder BUT patients (more than 60 years) were higher than in younger patients (*P* ⩽ 0.001). The mean Cys-C level of patients with PFL < 2.88 g/L was significantly lower than that of patients with PFL ≥ 2.88 g/L (*P* = 0.023). Even more, the levels of serum Cys-C in male BUT patients were higher than that in female patients (*P* = 0.012). A lot of research suggests that age-related reductions in the glomerular filtration rate (GFR) [25] lead to age-dependent increases in the concentrations of serum Cys-C [26, 27]. But in our study, in the group of GFR ≥ 90 mL/min/1.73 m^2^, the concentrations of serum Cys-C also have an age-dependent increase, indicating that age-dependent increases in the concentrations of serum Cys-C just partly depended on age-related reductions in the glomerular filtration rate (GFR). Using linear correlation analyses, there were positive correlations between circulating Cys-C levels, age (*r* = 0.3221, *P* = 0.0001), and plasma fibrinogen levels (*r* = 0.1665, *P* = 0.028). Sex is also an independent factor affecting the concentrations of serum Cys-C excluding the effect of glomerular filtration rate (GFR) decline.

There were insignificant associations between the levels of serum Cys-C and clinical characteristics, such as smoking history and painless macroscopic hematuria. BUC is one of the most common malignancies in the industrialized world, mainly caused by smoking and occupational exposure to chemicals. The favorable prognosis of early stage bladder cancer underscores the importance of early detection for the treatment of this disease. The high recurrence rate of this malignancy also highlights the need for close postdiagnosis monitoring of bladder cancer patients [28].

In conclusion, no statistically significant differences in the levels of serum Cys-C were found among the tumor characteristics (tumor number, tumor size, and pathological characteristics (all *P* > 0.05)). Circulating Cys-C was not a potential marker for BUC tumorigenesis and was not a reliable predictor for clinicopathological characteristics of BUC patients.

## Figures and Tables

**Table 1:** Clinical characteristics of patients with BUT.

GFR (mg/min/1.73 m^2^)	Number of patients (*n*, %)			Cys-C (mg/L), mean ± SD	*P*
	Male	Female	Total		
≥90	263 (61.88)	58 (13.65)	321 (75.53)	0.91 ± 0.15	0.001
<90	81 (19.06)	23 (5.41)	104 (24.47)	1.32 ± 0.77	

GFR: glomerular filtration rate; Cys-C: cystatin C; P: Student's *t*-test.

**Table 2:** The level of serum Cys-C in whole BUT patients.

Parameters	Number of patients	Cys-C	*P*
		levels (mg/L, mean ± SD)	
Age			**0.013**
<60 yr.	167	0.95 ± 0.57	
≥60 yr.	258	1.05 ± 0.31	
Sex			0.704
Male	344	1.02 ± 0.45	
Female	81	1.00 ± 0.37	
Smoking history			0.153
Never	277	0.99 ± 0.30	
Ever	148	1.05 ± 0.62	
Painless macroscopic hematuria			0.928
No	89	1.00 ± 0.34	
Yes	336	1.01 ± 0.46	
WBC count^a^			0.553
<6.1 × 10^9^/L	209	1.03 ± 0.56	
≥6.1 × 10^9^/L	216	1.00 ± 0.27	
PLT count^a^			0.525
<213 × 10^9^/L	212	1.03 ± 0.33	
≥213 × 10^9^/L	213	1.00 ± 0.52	
PFL^a^			**0.030**
<2.9 g/L	200	0.95 ± 0.25	
≥2.9 g/L	224	1.07 ± 0.55	
Tumor number			0.095
Single	291	0.98 ± 0.25	
Multiple	134	1.08 ± 0.68	
Tumor size^a^			0.378
<2 cm	124	0.98 ± 0.29	
≥2 cm	299	1.02 ± 0.49	
Pathological characteristics			0.156
Papilloma	27	0.94 ± 0.18	
PUNLMP	67	0.931 ± 0.16	
BUC	331	1.03 ± 0.48	
Grade			0.836
Low-grade	180	1.03 ± 0.60	
High-grade	151	1.04 ± 0.29	
Stage			0.783
Ta	178	1.03 ± 0.59	
T1	91	1.05 ± 0.37	
T2	34	0.98 ± 0.20	
T3	17	0.97 ± 0.22	
T4	11	1.18 ± 0.32	

Continuous variables are expressed as median ^a^. Bold values are statistically significant (*P* < 0.05). PUNLMP: papillary urothelial neoplasm of low malignant potential; BUC: bladder urothelial carcinoma; WBC: white blood cell; PLT: platelet; PFL: plasma fibrinogen levels.

**Table 3:** The level of serum Cys-C in BUT patients with normal GFR.

Parameters	Number of patients	Cys-C	*P*
		levels (mg/L, mean ± SD)	
Age			**0.001**
<60 yr.	144	0.88 ± 0.15	
≥60 yr.	177	0.94 ± 0.14	
Sex			**0.012**
Male	263	0.92 ± 0.14	
Female	58	0.87 ± 0.15	
Smoking history			0.233
Never	204	0.91 ± 0.15	
Ever	117	0.93 ± 0.15	
Painless macroscopic hematuria			0.101
No	59	0.89 ± 0.13	
Yes	262	0.92 ± 0.15	
WBC count^a^			0.149
<6.08 × 10^9^/L	160	0.90 ± 0.14	
≥6.08 × 10^9^/L	161	0.93 ± 0.15	
PLT count^a^			0.568
<216 × 10^9^/L	160	0.92 ± 0.15	
≥216 × 10^9^/L	161	0.91 ± 0.14	
PFL^a^			**0.023**
<2.88 g/L	161	0.90 ± 0.15	
≥2.88 g/L	160	0.93 ± 0.15	
Tumor number			0.541
Single	229	0.92 ± 0.13	
Multiple	92	0.91 ± 0.16	
Tumor size^a^			0.619
<2 cm	101	0.91 ± 0.15	
≥2 cm	218	0.92 ± 0.15	
Pathological characteristics			0.733
Papilloma	21	0.89 ± 0.17	
PUNLMP	60	0.91 ± 0.14	
BUC	240	0.92 ± 0.15	
Grade			0.390
Low-grade	141	0.91 ± 0.14	
High-grade	99	0.93 ± 0.15	
Stage			0.913
Ta	140	0.92 ± 0.79	
T1	62	0.92 ± 0.16	
T2	27	0.90 ± 0.13	
T3	7	0.87 ± 0.20	
T4	4	0.90 ± 0.08	

Continuous variables are expressed as median ^a^. Bold values are statistically significant (*P* < 0.05). PUNLMP: papillary urothelial neoplasm of low malignant potential; BUC: bladder urothelial carcinoma; WBC: white blood cell; PLT: platelet; PFL: plasma fibrinogen levels.
